# Telemedicine in Swedish primary health care - a web-based survey exploring patient satisfaction

**DOI:** 10.1186/s12913-023-09133-z

**Published:** 2023-02-08

**Authors:** Carl Rockler Meurling, Elisabet Adell, Moa Wolff, Susanna Calling, Veronica Milos Nymberg, Beata Borgström Bolmsjö

**Affiliations:** Department of Clinical Sciences Malmö, Center for Primary Health Care Research, Clinical Research Centre (CRC), Lund University, building 28, floor 11, Jan Waldenströms gata 35, Skåne University Hospital, SE-205 02 Malmö, Region Skåne Sweden

**Keywords:** Telemedicine, Digital care, Satisfaction, Primary health care, Symptom group

## Abstract

**Background:**

Direct-to-consumer telemedicine (TM), with patients having access to a physician via video or text chat, has gradually been introduced into Swedish primary care during the last two decades. Earlier studies have concluded that patients were generally satisfied with TM and the satisfaction rate was consistently above 80% and comparable with in-person visits. Despite the number of studies looking at user experience, studies assessing what factors influence patient satisfaction are lacking. To further develop digital care, it is important to explore the patients’ opinions of this relatively new phenomenon. The primary aim of this study was to explore patient opinions regarding satisfaction with TM-provided care, based on different age groups, sex, symptoms, and which type of health care professional they were assessed by.

**Methods:**

The study was a quantitative web survey performed in 2020–2021 in which 688 patients using a TM provider in Southern Sweden responded. The results were analysed using Chi-2 test with the main outputs; satisfaction level and percentage that would use TM for similar symptoms in the future.

**Results:**

The results from the survey population show that patients that were assessed by a doctor were more likely to intend to use TM in the future and were more satisfied with the visit than patients assessed by a nurse. Our results also show that patients older than 70 years of age were less likely to use TM again compared to the total population.

**Conclusion:**

This study shows that patient satisfaction with TM varied depending on the patient’s age. The older patients were less satisfied than their younger equivalents. For patient satisfaction to be high, it was also crucial which health care provider the patient had been assessed by. The patients were more satisfied when assessed by a doctor than by a nurse. In addition, the study shows that patient satisfaction with TM was generally comparable to traditional care.

**Supplementary Information:**

The online version contains supplementary material available at 10.1186/s12913-023-09133-z.

## Background

Direct-to-consumer telemedicine (TM), with patients having access to a physician via video or text chat, has gradually been introduced in Sweden during the last two decades. TM has been provided both through private companies and public Primary Health Care Centres (PHCC) using digital platforms [1]. In the Swedish region of Skåne, the public TM provider “Primärvården Skåne online” (PVO) was introduced in 2019 as a digital platform for primary health care patients. Prior to the introduction of PVO, Skåne’s 1.4 million inhabitants had access to TM through private companies for several years.

During the Covid-19 pandemic, public access to health care worldwide was remodelled at a fast pace. Prior to the pandemic, more than two-thirds of Swedish physicians did not communicate with patients via e-mail, video consultations or other TM interaction tools in their daily clinical work [2]. The need to keep physical encounters to a minimum during the pandemic increased the focus on TM worldwide. In the early stages of the pandemic, patients were reluctant to visit their PHCC and PHCC strived to develop alternatives to physical encounters [3]. Therefore, the number of TM consultations increased during the pandemic [4].

The usage of TM in primary health care has been controversial both in Sweden and worldwide with political and social debates regarding costs vs effectiveness [5]. A report performed by the Swedish government’s official investigations concluded that Swedish Health Care could potentially save up to 25% of its annual costs If they implemented more digital visits [6], although these figures have been largely questioned [7, 8]. The evidence regarding the economic savings of TM in health care is therefore limited.

A systematic review from 2021 concerning Automated Digital Triage in Primary Care concluded that there were no relevant studies published regarding public health economic advantages and neither any controlled studies regarding patient-related outcomes [9].

A pre-pandemic study from Sweden concluded that TM visits for respiratory or urinary symptoms resulted in a larger proportion of physical revisits in the first 48 hours after the TM visit but similar utilization of physical visits the following 2 weeks, compared to patients with an initial physical visit [10]. Another Swedish study concluded that only a few TM consultations led to further physical contact and that the general users were satisfied with their visit [4]. Despite the number of studies looking at user experience, relevant studies assessing what factors influence patient satisfaction are lacking.

To further develop and successfully implement digital care, it is important to explore the patients’ opinions of this relatively new phenomenon. Furthermore, it is important to investigate which patients and what needs can be met - or not met - with digital care. Therefore, the primary aim of this study was to explore patients’ satisfaction with TM provided care, based on different age groups, sex, symptoms, and which type of health care professional they were assessed by.

## Methods

### Design and participants

PVO was launched in November 2019 in the Swedish region of Skåne as a digital platform for primary health care patients with the possibility to be assessed either by a nurse and/or a doctor. Patients could contact the platform online and register using their unique Bank-ID authentication. After the registration, they completed a digital symptom checker that gave the triage nurse detailed information about the main complaint and the patient’s expectations with the TM visit. Some patients were automatically directed to a doctor. For example, if there was a need for a prescription or a sick leave certificate. The patients directed to the nurse were triaged to either self-care, medical counselling by a nurse or contact with a doctor for further assessment. Between September 23rd 2020 and November 23rd 2021 (including a gap when the web survey was down between January 11th 2021 and April 29th 2021), a total of 11,825 patients used PVO and were asked to anonymously fill in a non-mandatory web-based questionnaire after they had completed their online consultation. Our study analysed the outcome of these questionnaires and addressed the questions related to patient satisfaction.

### Questionnaire design

The survey was created in the program Research Electronic Data Capture (RedCap) [11] The survey intended to investigate whether PVO could relieve ordinary primary health care, how satisfied the patients were, if they were likely to use the service again and if there were differences in satisfaction depending on the symptom group that the patient contacted the PVO for. The symptom groups were defined as; skin-related, upper respiratory, urinary tract, prescription renewal, lower abdomen, eye, wound injury and other (Additional file 1).

In the first part of the survey, there were four questions with multiple choice answers including sex, age, reasons for contacting PVO in the predefined symptom groups and what health care personnel the patient was assessed by. Thereafter there were four questions with yes/no/do not know answers regarding if the patient had been assessed by any other health care provider for the same issue during this symptom period before the contact with PVO that day, if the patient would have visited another health care provider the same day if they had not got the PVO appointment, if the patient after this assessment by PVO would still seek another health care provider for the same issue within a week, and lastly if the patient would use PVO again for similar symptoms in the future. The final question in the survey was a scale between 0 and 100 where the patient could rate how likely it was that he/she would recommend PVO to others. In addition, there was a possibility for the patients to leave voluntary comments (Additional file 1).

### Outcomes

Primary outcomes were the results from the questions “Would you visit PVO for similar symptoms in the future” and “How likely are you to recommend PVO to others”. If the patient answered yes to the first question, the patient was regarded as satisfied. As the latter question was answered on a scale from 0 to 100 where 100 was very likely, the results from this question are referred to as satisfaction rate further on. The results were further divided into subgroups for sex, age, assessed by nurse or doctor and symptom group. The secondary outcomes were the results of the other questions in the survey.

### Data analysis

Data were downloaded to Excel and manually processed and validated. All data were then analysed using SPSS version 28.0.1.

To determine statistically significant differences between symptom groups and sex, age groups, assessed by doctor or nurse as well as the yes/no/do not know questions, chi-2 test was used with a level of significance at 0.05.

Regarding the answers to the question “would you visit PVO for similar symptoms in the future” we compared the yes/no/do not know results for the different subgroups with the answers in the total survey population. As above, the determination of significant differences was assessed by chi-2 test with a level of significance at 0.05. Furthermore, after excluding the undecided respondents (do not know), a yes/no ratio for each subgroup (sex, age, symptom, assessed by) was calculated. The ratio was then assessed for different subgroups compared to the ratio of the total survey population.

To determine the associations for the ratio yes/no to the question “would you visit PVO for similar symptoms in the future, we conducted a binary logistic regression analysis with type of caregiver (doctor/nurse) as a dependent variable. A backwards stepwise selection model was applied for the covariates age, sex, and symptom groups.

## Results

**Fig. 1:**
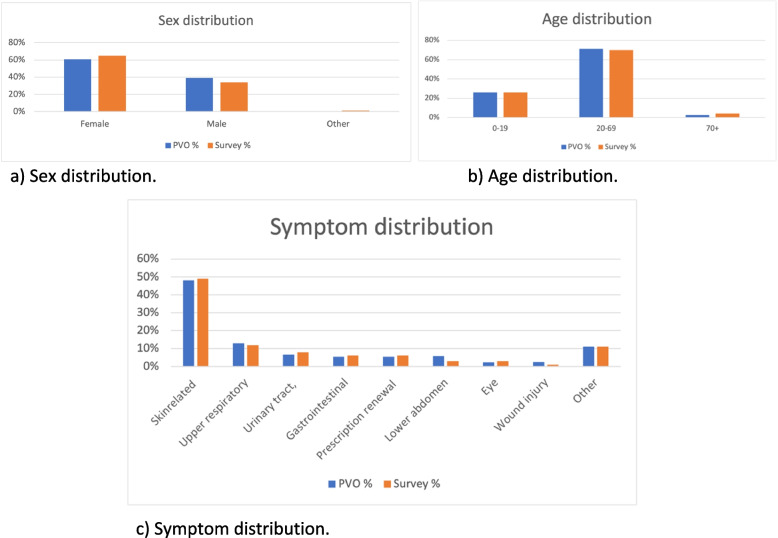
**a**-**c** Distribution of actual PVO visits compared to survey response population

The survey was completed by 688 PVO patients. As the surveys from 18 of the 688 respondents were lacking significant information, they were excluded from the study thus leading to 670 valid responses. The survey population consisted of 433 women, 231 men and 6 with the definition of sex as “other”. Regarding age groups, 180 of the respondents were between 0 and 19 years and 25 persons were over the age of 70 (Table 1). During the period when the web survey was accessible, a total of 11,825 patients used the service, leading to a response rate of 5.8%. The total PVO population included 61% women, which corresponded to the survey population of 65% women (Fig. 1a). In the total PVO population, 26% were between 0 and 19 years and 3% were over 70 years of age. The young age group corresponded well to the survey population where 26% were between 0 and 19 years. The older age group was a bit more responsive and accounted for 4% of the survey population (Fig. 1b). The symptom groups also correlated well between the PVO population and the survey population (Fig. 1c).

**Table 1:**
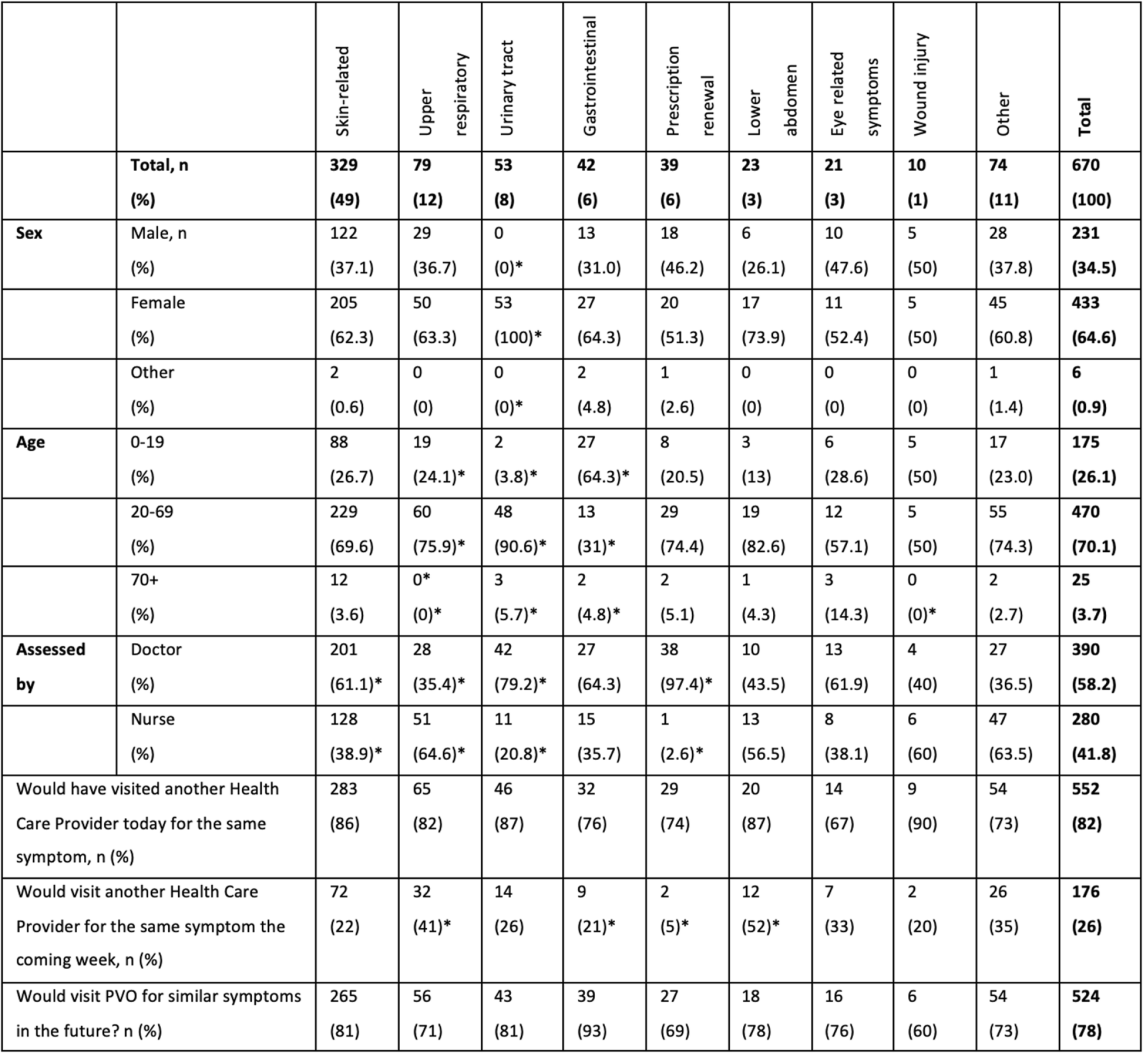
Description of patients categorised into symptom groups

*p-value < 0.05 compared to total study population

Women were in majority in all subcategories pending between 100% women for urinary tract symptoms to 50% for wound injury. The most common symptom for all age groups was skin-related issues, which made up 49% of all visits. The 0–19 age group was overrepresented for gastrointestinal symptoms and the 20–69 age group for urinary tract symptoms compared to the total study population (Table 1).

As seen in Table 1, 58% of all patients were assessed by a doctor while 42% were assessed exclusively by a nurse. Prescription renewals, urinary tract and skin-related symptoms were more likely to be handled by a doctor while upper respiratory symptoms were more likely to be handled by a nurse compared to the total study population.

**Fig. 2:**
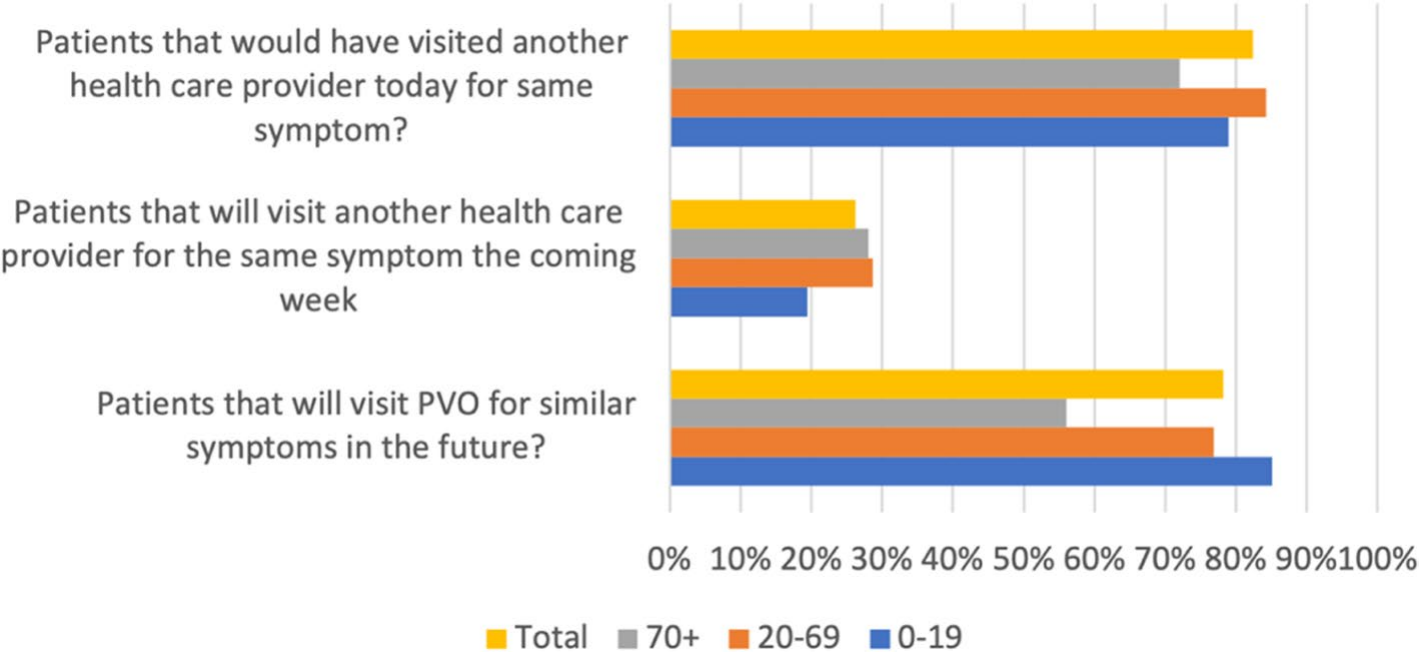
Age group responses to follow-up questions

As seen in Fig. 2, 82% of the respondents would have visited another health care provider if they had not got the PVO appointment that day. About three-quarters (74%) of the patients stated that they would not seek another health care provider for the same cause during the coming week, which indicated that their health problems were completely taken care of during the digital visit. Individuals with upper respiratory- and lower abdomen symptoms were more likely to consider visiting a health care provider the coming week and those with gastrointestinal symptoms and prescription renewals were less likely to consider visiting a health care provider the next week compared to the total study population (Fig. 2).

### Patient satisfaction

In total 78% of the patients (*n* = 524) would use PVO again for similar symptoms (answered yes), 11% would not use PVO again (answered no) and 11% were uncertain if they would use PVO again. The ratio of the answers yes/no was 7.1 in total (Table 2).

**Table 2:**
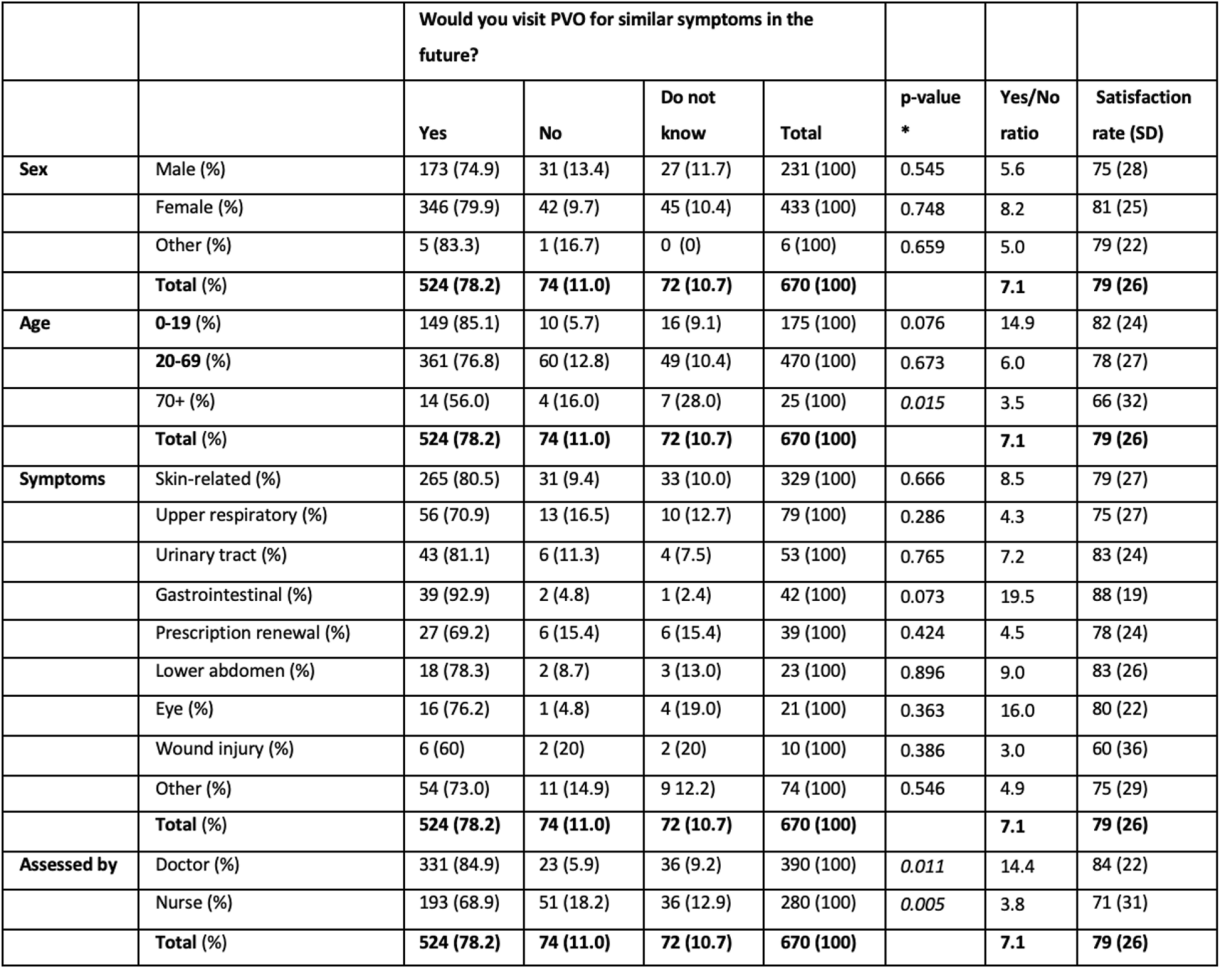
Patient satisfaction for different groups

*Based on Person Chi-Square comparing the yes/no/do not know results for the subcategories to the total survey population

The patients were asked on a scale between 0 and 100 how likely they were to recommend PVO to others and the mean rating was 79 with a standard deviation (SD) of 26.

### Sex

A trend of higher tendency to use PVO again and a higher satisfaction rate was seen among women, but there were no statistically significant differences between the sexes in total for any of the symptom groups.

### Age

The age group 0–19 had both the highest proportion of patients that would use PVO again and the highest satisfaction rate. On the contrary, the lowest satisfaction rate was found for the oldest age group 70+, which also had the lowest proportion of patients that would use PVO again. For the oldest age group, the ratio of patients that would use PVO again was significantly lower compared to the total population.

### Symptom group

The percentage of patients that would use PVO again varied between 93% for gastrointestinal symptoms to 60% for wound injury. Similarly, the satisfaction rate spanned between 88 (SD = 19) for gastrointestinal and 60 (SD = 36) for wound injury. None of the subgroups of symptoms showed any significant difference compared to the total study population.

### Nurse or doctor assessment

**Fig. 3:**
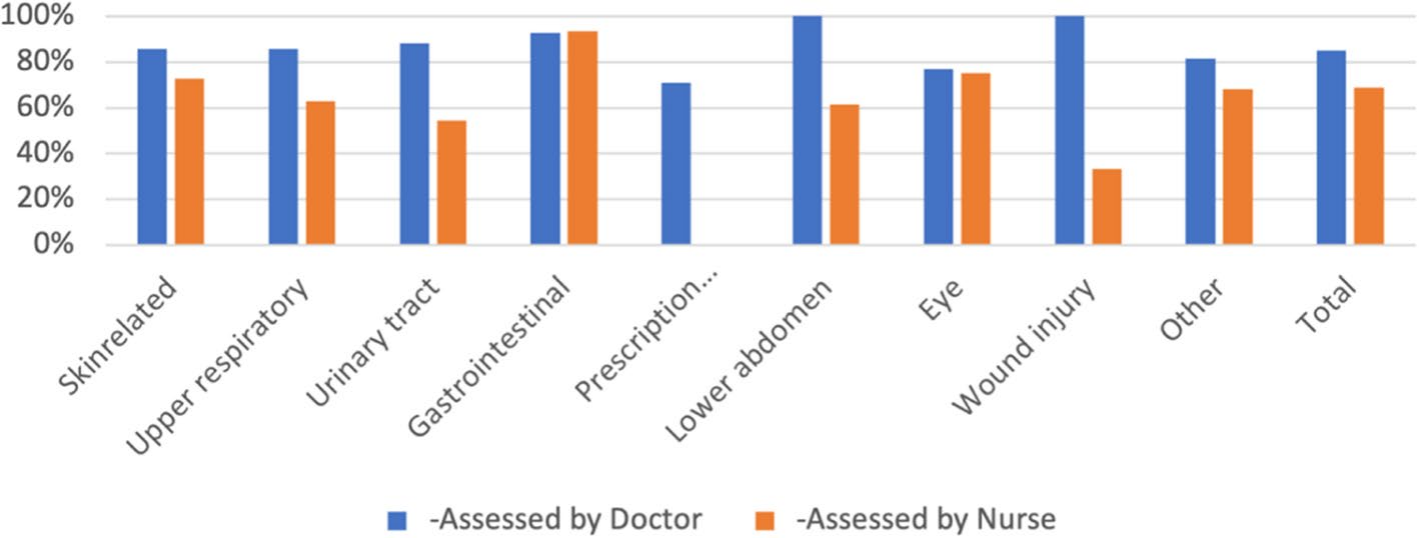
Percentage that would use PVO again grouped by symptom and health care professional

The percentage that would use PVO again (were satisfied) grouped by symptom showed that patients assessed by a doctor were more likely to use PVO again for all symptom groups (Fig. 3). The patients assessed by a nurse were significantly less satisfied than the patients assessed by a doctor (Table 2). This could also be seen in the satisfaction rate where patients assessed by a nurse rated 71 (SD = 31) and patients assessed by a doctor rated 84 (SD = 22), compared to the total population’s satisfaction rate of 79 (SD = 26). The “yes/no” ratio (satisfied/not satisfied) for doctors was 14.4 compared to 3.8 for nurses. Patients who were exclusively assessed by a nurse were significantly less likely to use PVO again and were also less satisfied. To ensure there were no cross interactions we did a binary logistic regression analysis of satisfaction level based on if the patient was assessed by a nurse or a doctor. The analysis showed that the statistically significant difference remained despite the inclusion of the covariates age, sex, and symptom group.

## Discussion

### Main findings

Our study shows that patient satisfaction for the survey population with the present TM service was fairly high with 78% of the respondents answering that they would use PVO for similar symptoms in the future and an overall satisfaction rate of 79 (0–100 scale). Factors significantly associated with high satisfaction were lower age and being assessed by a doctor instead of a nurse. Most of the respondents (82%) would have visited another health care provider if they had not gotten the PVO appointment the same day. About three-quarters (74%) of the respondents did not intend to seek more health care for the same cause during the coming week.

The high satisfaction with TM is in-line with physical care visits in previous research where around 80% would respond yes to the question if they would recommend their PHC to someone in a similar situation [12]. The oldest age group was less likely to use PVO again and they were also least satisfied with their visit. It has earlier been shown that personal interaction and continuity are more important for older patients than time or travel convenience and that they are not as familiar with describing symptoms in digital format as younger patients [13]. This could be the reason for a lower satisfaction level for the oldest age group.

Our results show that the survey respondents were more likely to use PVO again if assessed by a doctor compared to a nurse. Earlier research has described patients using TM as being confident in knowing that TM was the right level of care for them [4]. Therefore, one possible explanation is that the nurse’s assessment of patients in less need of health care is not in-line with the patient’s own perception. If users’ expectation of contact with a doctor is not met, this could affect the likelihood to use the service again [14]. The finding that patient satisfaction was lower for patients assessed by a nurse is opposite to the results from a Cochrane systematic review of 18 randomised trials in 2018. This showed that patient satisfaction in primary care was slightly higher for nurse-led care for patients with all types of health problems, excluding mental health problems, regarding both first contact and ongoing care [15]. This discrepancy between our findings that patient satisfaction when assessed by a nurse was lower in TM further emphasises the need for more research focused on TM. Besides the statistically significant findings, we found some trends in certain areas, but where further research is needed. It seemed like the respondents under the age of 20 were more likely to use PVO again than the rest of the population. Earlier research has shown that time and accessibility is important for parents with children in need of health care. By using TM, they can cut travel time and increase their flexibility [4]. We also noted differences between the symptom groups where the overall satisfaction level was the highest for gastrointestinal symptoms, which further suggests that certain subcategories are more suitable for TM.

### Strengths and limitations

One strength of the study is that age and sex distribution seem to be in-line with the overall demographic characteristics of patients visiting PVO during the timespan, indicating that the survey cohort is representative of the whole patient group.

Another strength is that the survey was accessible to patients during both summer and winter. In that way, we could explore patient satisfaction over a larger time span and not only during typically seasonal health issues.

A major limitation of this study is the low response rate. Web-based surveys are known to have a significantly lower response rate than paper-based [16]. A Danish randomised study showed a risk difference of 55.3% in response rate for the internet group compared to the paper-based for a survey distributed to patients with breast cancer. The response rate for the Danish web-based survey was 17.9%, which is a lot higher than ours but still considered to be low [17]. A reason for this may be that a cancer patient is more prone to answer questions about their treatment than a patient with a minor problem in primary care, and hence our response rate is even lower. Although, even web-based surveys to cancer patients may have as low response rate as 5.5% [18].

A way to increase the response rate would have been through reminders [19]. The design of this study did not enable this with the survey in a pop-up window not connected with patient data.

However, despite the low response rate, the evidence shows comparable or even better reliability with web-based surveys compared with paper-based surveys [20]. It is also possible that patients want to justify their digital visit by saying they would have sought another health care provider anyway [21]. The survey was intended to be short and easy to complete, although further and more detailed questions could have provided more knowledge to understand the patients’ satisfaction.

We do not believe that it is possible to ignore the nonresponse bias in our study. The mechanisms that result in nonresponse can only be speculated. As patients who are extremely positive/negative are more prone to answer surveys [22], one can believe that the respondents to the survey want to express something either positive or negative about their experience. The nonrespondents may have been more neutral in their responses. We are not able to measure the relationship between the opinions of the different groups, since the required data is unavailable. Our speculations about the nonresponders being more neutral in their opinions about the PVO service make us believe that the costly pursuit of a high response rate may had offered little or no reduction in nonresponse bias. There are statistical procedures to correct for the sampling bias introduced by nonresponse, but as stated by Burkell et al. [23], unfortunately these statistics work best when they are needed least: at low levels of nonresponse, and hence would not be possible to use for this material.

However, we do have almost 700 responses and we believe that these opinions are important to show, as we are dealing with a relatively new phenomenon of telemedicine. For further development of the service, it is important to acknowledge the respondents’ opinions.

A further limitation of this study is that it only covers one region of Sweden. The care delivered from PVO does though serve patients from multiple PHCCs and is provided to patients throughout the whole region, covering a rather large geographical area with a diverse population regarding sociodemographic and rural/urban determinants. However, in future studies, it would be beneficial to involve digital providers in other regions.

### Comparison with other studies

Several previous studies have focused on the impact of TM on accessibility, as well as staff and patient satisfaction. A systematic review from 2016 concluded that patients were generally satisfied with TM and the satisfaction rate was consistently above 80% and comparable with in-person visits [24].

The satisfaction rate of the digital visits of PVO was in-line with the results from the Swedish National Patient survey 2021 where 78.5% (*N* = 10,309) of respondents in Skåne would recommend their physical PHCC to someone in a similar health situation [12]. It is also consistent with previous research showing that satisfaction rates for TM have been comparable with in-person visits [24] and that users of TM were generally quite satisfied [4, 24].

Most of the patients were women, which is in-line with previous studies that have shown that women use TM more often than men [1, 4, 25]. Our results show that 82% would have used other health care providers if they would not have come in contact with PVO. These results are in-line with a study from 2018 showing 78% [26] would have used other health care providers if they had not used TM for the same cause. This indicates that TM may unburden physical health care.

A systematic review of 18 randomised trials from 2018 showed that patient satisfaction is probably slightly higher in physical nurse-led primary care than in doctor-led primary care [15]; this contradicts our main finding that patients were more satisfied when assessed by a doctor in TM care.

A study from the US in 2016 showed that the primary motivation for using telehealth was shorter waiting times [25]. One-third of the patients in this study - and particularly those with no health insurance - preferred a TM visit to a traditional visit [25]. Accessibility to primary health care could also have been important for our population but was not explored further in the survey, but since Swedish health care is largely governmentally funded, affordability would not have been a large issue for this survey population.

## Conclusion

The TM-users in southern Sweden who responded to the survey, showed user satisfaction at the same level as for physical care, and were seven times more likely to consider revisiting PVO for similar symptoms again than not using PVO again. The satisfaction was significantly lower among the age group over 70 years of age. We found a significantly lower likelihood to consider using PVO again for the patients that were solely assessed by a nurse.

The results from our study can be factored in when developing future digital care. Significant adjustments in digital care are needed for meeting the needs of elderly patients. It is also important to design a model of digital care where every professional role is used in the best way possible.

## Supplementary Information

**Additional file 1.**

## Data Availability

The datasets generated and analysed during the current study are not publicly available due to national regulations but are available from the corresponding author on reasonable request.

## Abbreviations

PHCC: Primary Health Care Centres
PVO: Primärvården Skåne online (a public digital platform for primary health care patients in the southern region of Sweden)
SD: Standard Deviation
TM: Telemedicine
