# Supplementary material for: Telemedicine in Swedish primary health care - a web-based survey exploring patient satisfaction
Source: BMC Health Serv Res. 2023 Feb 8;23:129. doi: 10.1186/s12913-023-09133-z (PMC9905766; doi:10.1186/s12913-023-09133-z)
Supplement: Supplementary file 1 — Additional file 1. [file 12913_2023_9133_MOESM1_ESM.docx]

**__________________________________________________________________________________**

**Survey PV Online**

Your answer is voluntary, but very valuable for gaining knowledge on how PV Online is perceived by the users.

Your answer is anonymous and can neither be connected to your visit nor to any other patient record.

Response was added on: (yymmdd) ____________________

1. Sex : Male Female Other

(Choose the alternative that applies to you, If you are a caregiver of a child, add the alternative that applies to the child)

1. Age: 0-3 years 4-12 years 13-19 years 20-29 years 30-39 years

40-49 years 50-59 years 60-69 years above 70 years

(Choose the alternative that applies to you, If you are a caregiver of a child, add the alternative that applies to the child)

1. Who helped you today? Nurse Doctor Nurse and Doctor Don’t know

4 . What problem did you seek help for at today’s visit at PV Online?

- Cough/ nasal congestion/ sinus problems
- Gastrointestinal problems
- Eye-related symptoms
- Wound injury
- Skin-related symptoms
- Urinarytract symptoms
- Lower abdomen symptoms
- Prescription renewal
- Other

5. Have you visited any other health care provider today or the last week for the same symptom? (i.e. a primary health care center or the emergency department)

Yes No Don’t know

6. If you hadn’t visited PV Online today, would you instead have visited any other health care provider for the same symptom?

Yes No Don’t know

7. After this PVO appointment, will you visit any other health care provider for the same symptom the coming week?

Yes No Don’t know

8. Will you visit PV Online for similar symptoms in the future?

Yes No Don’t know

9. Rate how likely it is that you would recommend PVO to others:

Very unlikely Very likely

0 ___________________________________________________________________________100

10. Free comments :

*Thank you for your participation!*
